# TNF-α Affects Signature Cytokines of Th1 and Th17 T Cell Subsets through Differential Actions on TNFR1 and TNFR2

**DOI:** 10.3390/ijms23169306

**Published:** 2022-08-18

**Authors:** Bárbara Pesce, Carolina H. Ribeiro, Milton Larrondo, Verónica Ramos, Lilian Soto, Diego Catalán, Juan Carlos Aguillón

**Affiliations:** 1 Immune Regulation and Tolerance Research Group (IRTGroup), Programa Disciplinario de Inmunología, Instituto de Ciencias Biomédicas (ICBM), Facultad de Medicina, Universidad de Chile, Santiago 8380453, Chile; 2 Laboratorio MED.UCHILE-FACS, Instituto de Ciencias Biomédicas (ICBM), Facultad de Medicina, Universidad de Chile, Santiago 8380453, Chile; 3 Hospital Clínico, Universidad de Chile, Santiago 8380453, Chile

**Keywords:** tumor necrosis factor (TNF)-α, TNF-α receptors (TNFR), Th1 cells, Th17 cells, rheumatoid arthritis (RA)

## Abstract

Tumor necrosis factor (TNF)-α is a pleiotropic cytokine implicated in the etiology of several autoimmune diseases, including rheumatoid arthritis (RA). TNF-α regulates diverse effector functions through the activation of TNF-α receptor (TNFR)1 and TNFR2. Although the detrimental role of this cytokine has been addressed in distinct disease settings, the effects of TNF-α on cytokine production by isolated CD4^+^ T helper type 1 (Th1) and Th17 cells, two T cell subpopulations that contribute to the pathogenesis of RA, have not been completely elucidated. Here, we show that TNF-α promotes a reduction and expansion in the frequency of both T cell subsets producing IFN-γ and IL-17, respectively. Selective blockade of TNFR1 or TNFR2 on Th1 and Th17 cells revealed that TNFR2 mediates the decrease in IFN-γ production, while signaling through both receptors augments IL-17 production. We also demonstrate that Th1, but not Th17 cells from RA patients present lower levels of TNFR1 compared to healthy controls, whereas TNFR2 expression on both T cell types is similar between patients and controls. Since TNF-α receptors levels in RA patients are not significantly changed by the therapeutic blockade of TNF-α, we propose that targeting TNFR2 may represent an alternative strategy to normalize the levels of key cytokines that contribute to RA pathogenesis.

## 1. Introduction

It has long been established that the activated subsets of CD4^+^ T helper type 1 (Th1), Th2, and Th17 lymphocytes are classified according to their selective cytokine production, expression of specific transcription factors that control lineage development, and expression of characteristic cell surface markers and chemokine receptors, besides exhibiting key effector functions in protective and abnormal immune responses [1]. Th1 cells are chemokine receptor CXCR3^+^ lymphocytes bearing the T-box expressed in T cells (T-bet) transcription factor, secrete interferon (IFN)-γ, tumor necrosis factor (TNF)-α and interleukin (IL)-2, and eradicate intracellular pathogens; conversely, Th2 lymphocytes express GATA-3 transcription factor and the chemokine receptor CCR4, secrete IL-4, IL-5, IL-9, and IL-13, and are involved in protection against parasitic infections and in allergic disorders [1,2]. In turn, Th17 lymphocytes express the retinoic acid-related orphan receptor (ROR) γt transcription factor; they also express CD161 and CCR6 and produce mainly IL-17A and IL-17F, as well as IL-21, IL-22, and TNF-α [3]. Although Th17 cells support the integrity of the intestinal barrier and participate in host defense against extracellular pathogens of fungal or bacterial origin [4,5], they also play an important role in the pathogenesis of chronic inflammation and autoimmune diseases, suggesting a dual function of these cells in host immunity [6].

Rheumatoid arthritis (RA) is a systemic and disabling autoimmune disease characterized by synovitis that causes articular cartilage and bone destruction [7]. Based on the chronic inflammatory conditions associated with this disease, it is now clear that the cytokine milieu plays a key role in RA pathogenesis [8]. Although RA had been initially associated with a Th1 polarized response at the site of inflammation [9], studies in humans and mice have demonstrated that both, IL-17 and IFN-γ contribute to its pathogenesis. On one hand, IL-17 induces the production of TNF-α and IL-1β by macrophages [10] and synergizes with these cytokines to increase IL-6 and IL-8 production by synoviocytes [11,12], which, along with other pro-inflammatory mediators, promote cartilage damage [13]. On the other hand, IFN-γ-producing cells have been detected in the inflamed synovium of RA patients [14], and serum levels of IFN-γ have been associated with disease severity [15]. Nevertheless, a protective role for IFN-γ in RA progression has been proposed, since this cytokine inhibits TNF-α-dependent proliferation of synoviocytes, collagenase production, granulocyte-macrophage colony-stimulating factor (GM-CSF) secretion, and neutrophil migration into the joints [16]. Although IFN-γ prevents Th17 cell development [17], a subset of IL-17-secreting cells within the inflamed joint exhibit a phenotype combining Th17 and Th1 cell features, secreting IFN-γ and expressing both, RORγt and T-bet transcription factors, hence their denomination as Th17/Th1 cells [18,19]. Therefore, a potential therapeutic intervention on cytokine production by Th17, Th1, and/or Th17/Th1 cells within the inflamed synovium may have a decisive impact in the course of the disease.

TNF-α is a pro-inflammatory cytokine that is abundantly present in the inflamed synovium, where it is produced by macrophages, fibroblasts, lymphocytes, and endothelial cells [20]. In RA, it mediates synovial inflammation and tissue destruction, since it is a potent inducer of proteases and pro-inflammatory cytokines and chemokines, such as IL-6 and IL-8, respectively, as well as adhesion molecules, thus promoting leukocyte chemoattraction and lymphoid migration into inflamed joint tissue [21]. Data sustaining a pivotal role for TNF-α in RA pathology in humans have arisen from several studies showing that the therapeutic blockade of TNF-α with neutralizing antibodies, such as infliximab and adalimumab, provides significant clinical benefit for RA patients [22].

TNF-α binds to two transmembrane receptor molecules, TNF-α receptor (TNFR)1 (p55) and TNFR2 (p75). While TNFR1 is expressed at low levels in a wide variety of cell types and tissues, TNFR2 expression is inducible on endothelial and immune cells, such as monocytes, macrophages, natural killer cells, and T and B lymphocytes [23]. TNFR2 is predominantly activated by membrane-bound TNF-α, whereas both, soluble and membrane TNF-α can equally activate TNFR1 [24]. Previous studies have demonstrated that TNFR1 and TNFR2 mediate diverse effector functions since their intracellular domains activate different signaling cascades. Upon TNF-α binding, TNFR1 initiates pro-inflammatory responses and apoptosis, as it presents an intracellular death domain motif. Conversely, TNFR2, which does not possess an intracellular death domain, triggers cell survival and proliferation [25].

In the synovium of active RA patients, both TNF-α receptors are upregulated, at the protein and mRNA levels [26]. In addition, in peripheral blood mononuclear cells (PBMCs) from RA patients, higher levels of TNF-α and TNFR1 mRNA have been detected, as compared to healthy donors, although TNFR2 mRNA levels were similar between patients and controls [27]. On one hand, TNFR1 has been regarded as the main responsible for the detrimental effects of TNF-α in RA [28], as TNFR1-deficient mice display reduced development of collagen-induced arthritis (CIA) [29]. Accordingly, engagement of TNFR1 promotes bone destruction by enhancing the generation of osteoclasts [30]. On the other hand, TNFR1 activation inhibited the development of pathogenic Th17 cells in CIA mice, while blockade of TNF-α concomitantly increased the numbers of Th1 and Th17 cells in draining lymph nodes and reduced the numbers of these cells in the joints [31]. As for TNFR2, mice that lack this receptor develop aggravated arthritis and joint destruction [32]; in addition, TNFR2-deficient mice can also present enhanced osteoclastogenesis, which worsens TNF-α-mediated arthritis [33]. Therefore, these differential effects of TNF-α on its receptors warrant further investigation for a deeper understanding of the roles these molecules play in the pathogenesis of RA.

Although the effector functions of TNF-α and TNF-α blockade have been extensively approached in several disease settings, the effect of TNF-α neutralization or TNF-α receptors blockade on cytokine production by circulating CD4^+^ T cell subpopulations remained to be evaluated. Here, we sought to determine the production of IFN-γ and IL-17 by TNF-α-stimulated peripheral Th1 and Th17 cells upon selective blockade of TNF-α, TNFR1, or TNFR2. Our results show evidence for a differential effect of TNF-α on TNFR1 and TNFR2-mediated cytokine production by both T cell subtypes, which may have an impact on RA outcome.

## 2. Results

### 2.1. TNF-α Reduces IFN-γ and Increases IL-17 Production by Th1 and Th17 Cells

**Figure 1:**
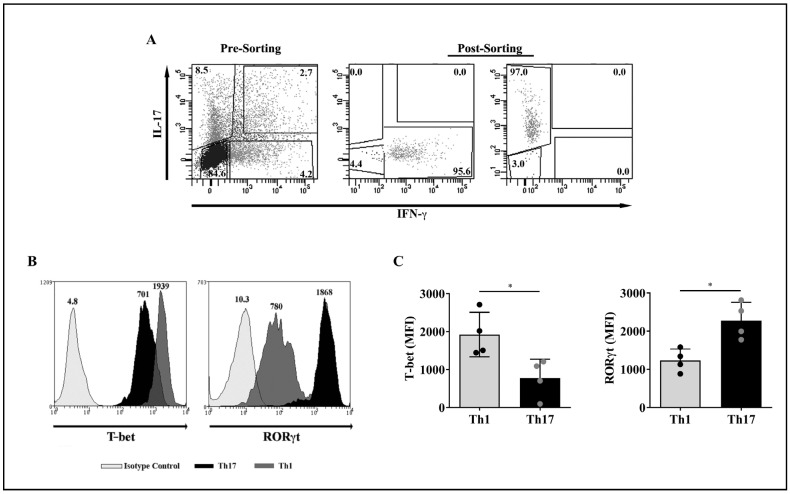
Phenotypic characterization of purified peripheral blood Th1 and Th17 cell subpopulations by flow cytometry. (**A**) A polyclonal stimulus of RosettSep™ kit-enriched CD4^+^ T lymphocytes with PMA and ionomycin plus anti-CD28 and anti-CD49d mAbs (pre-sorting) was followed by cell sorting of T cell subtypes based on cytokine production: IFN-γ for Th1 cells and IL-17 for Th17 cells (post-sorting). The numbers in each quadrant represent the percentage of cells. (**B**) Representative histograms showing the expression levels of the transcription factors T-bet and RORγt on purified Th1 and Th17 cells from a healthy donor. Light grey histograms, isotype control; grey histograms, Th1 cells; black histograms, Th17 cells. The numbers on top of each curve represent the mean fluorescence intensity (MFI) values. (**C**) MFI values of T-bet and RORγt levels on purified Th1 and Th17 cells obtained from four healthy donors. Bars represent the mean values ± standard deviation (SD). The Mann-Whitney test was used for statistical analysis. * *p* < 0.05.

We used PBMCs obtained from the buffy coats of healthy donors to enrich CD4^+^ T cells using a RosettSep™ kit (gating strategy shown in Supplementary Figure S1). Further, after a polyclonal stimulus of CD4^+^ T cells with phorbol 12-myristate 13-acetate (PMA) and ionomycin plus anti-CD28 and anti-CD49d monoclonal antibodies (mAbs), we purified Th1 and Th17 lymphocytes by cell sorting based on their signature cytokines, IFN-γ and IL-17, respectively, applying a cytokine capture assay. Purification of CD4^+^IFN-γ^+^ (Th1) and CD4^+^IL-17^+^ (Th17) cells yielded >95% enrichment (Figure 1A). To confirm cell purity, the expression of T-bet and RORγt on sorted T cell lineages was determined by flow cytometry. Figure 1B shows representative histograms that revealed the co-expression of T-bet and RORγt on both Th1 and Th17 cells. Nevertheless, results obtained from 4 healthy individuals, and indicated as mean fluorescence intensity (MFI) values, demonstrate that the levels of T-bet were significantly higher in Th1 compared to Th17 cells, while RORγt levels were significantly elevated in Th17 relative to Th1 cells (Figure 1C).

**Figure 2:**
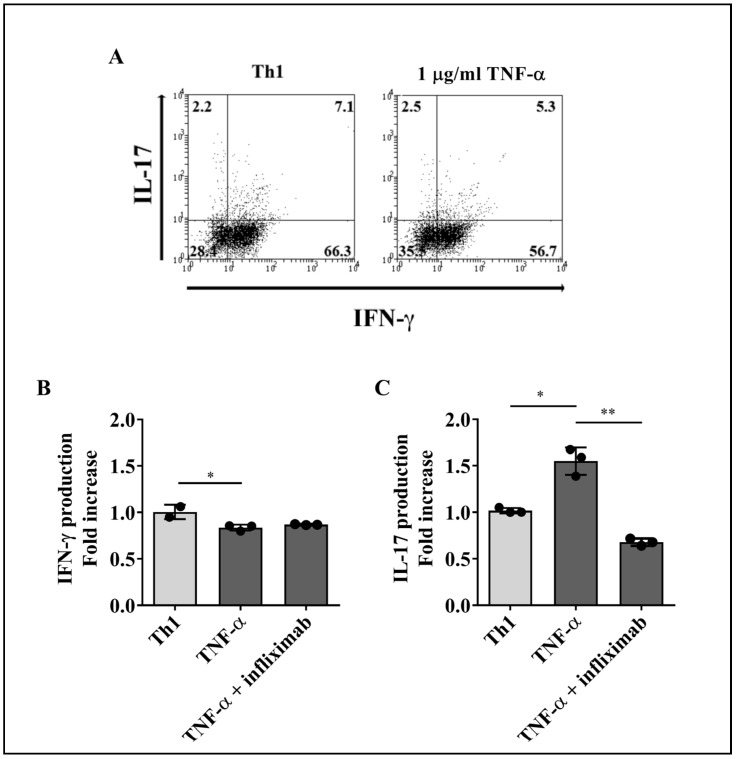
Effect of TNF-α on IFN-γ or IL-17 production by Th1 lymphocytes. Sorted Th1 cells were cultured for 4 days with 1 µg/mL of recombinant human TNF-α alone or in the presence of an anti-TNF-α blocking mAb (infliximab). Cells were then stimulated with PMA and ionomycin plus brefeldin A for 5 h and analyzed for cytokine production by flow cytometry. (**A**) Representative dot plots of Th1 cells upon stimulus with TNF-α. The numbers in each quadrant represent the percentage of cells. The effect of TNF-α on IFN-γ (**B**) or IL-17 (**C**) production was measured on Th1 cells from three healthy donors. Bars represent the mean values ± SD. Data were normalized against cells that did not receive TNF-α or TNF-α plus anti-TNF-α treatment. Kruskal–Wallis test followed by Dunn’s multiple comparison test was carried out to analyze differences between groups. * *p* < 0.05, ** *p* < 0.01.

**Figure 3:**
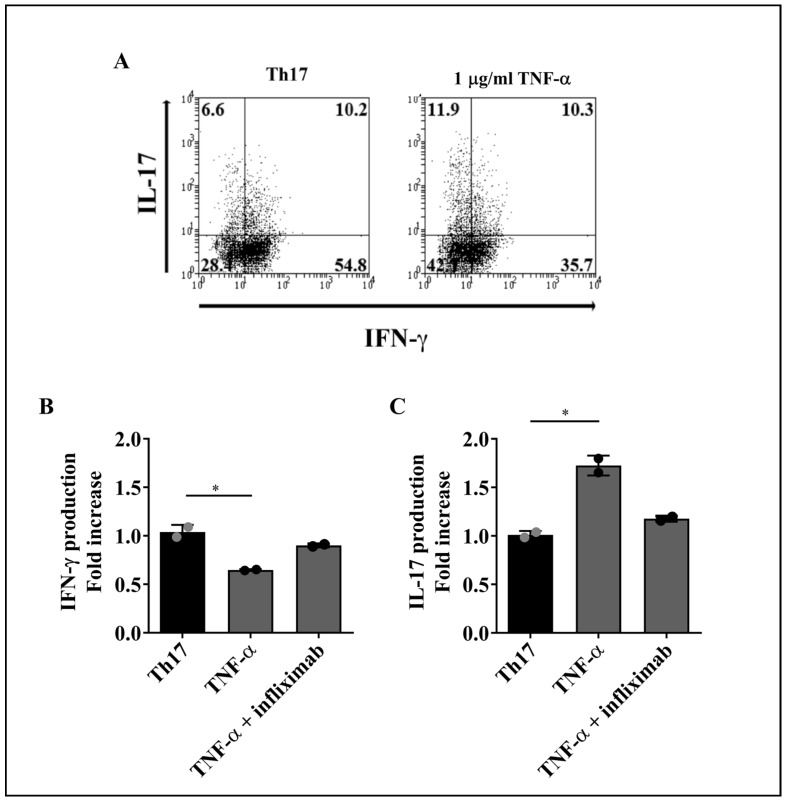
Effect of TNF-α on IFN-γ or IL-17 production by Th17 lymphocytes. Sorted Th17 cells were cultured for 4 days with 1 µg/mL of TNF-α alone or in the presence of an anti-TNF-α blocking mAb (infliximab). Cells were then stimulated with PMA and ionomycin plus brefeldin A for 5 h and analyzed for cytokine production by flow cytometry. (**A**) Representative dot plots of Th17 cells upon stimulus with TNF-α. The numbers in each quadrant represent the percentage of cells. The effect of TNF-α on IFN-γ (**B**) or IL-17 (**C**) production was measured on Th17 cells from two healthy donors. Bars represent the mean values ± SD. Data were normalized against cells that did not receive TNF-α or TNF-α plus anti-TNF-α treatment. Statistical analyses were performed with Kruskal-Wallis followed by Dunn’s multiple comparison tests. * *p* < 0.05.

To evaluate the effect of TNF-α on the production of IFN-γ and IL-17 by T cell subsets, purified lymphocytes were treated with recombinant human TNF-α in the presence or absence of a blocking anti-TNF-α mAb (infliximab) for 4 days, followed by stimulation with PMA and ionomycin plus brefeldin A to induce cytokine production. Increasing concentrations of TNF-α (0, 10, 100, and 1000 ng/mL) were used to stimulate Th1 and Th17 cells. As TNF-α at 1000 ng/mL consistently induced a response on both T cell subsets (Supplementary Figure S2), we decided to use this amount of cytokine (1 µg/mL) for our subsequent assays. The percentages of intracellular IFN-γ or IL-17-producing Th1 and Th17 cells were determined by flow cytometry, as illustrated by the representative dot plots shown in Figure 2A, for Th1, and Figure 3A, for Th17 cells.

Due to the interindividual variability often observed in human studies, flow cytometric data from cells treated with TNF-α or TNF-α plus infliximab were normalized relative to an internal control, which consisted of data obtained from cells that did not receive cytokine stimulus. Compared with cells that received PMA and ionomycin stimulus only, treatment with TNF-α induced a significant decrease in the percentages of Th1 and Th17 cells producing only IFN-γ^+^, while the baseline levels of IFN-γ in both T cell subsets did not change in the presence of TNF-α plus infliximab (Figure 2B and Figure 3B, respectively). Conversely, TNF-α promoted a significant expansion of Th1 and Th17 cells producing only IL-17 as compared to untreated cells, whereas TNF-α blockage restored the frequencies of IL-17^+^Th1 and IL-17^+^Th17 lymphocytes to the levels observed without TNF-α stimulus (Figure 2C and Figure 3C, respectively).

Altogether, these results indicate that, as for IFN-γ and IL-17 synthesis, TNF-α promotes a similar response in both, Th1 and Th17 cells. Moreover, TNF-α neutralization prevents TNF-α-induced alterations in IFN-γ and IL-17 production by both T cell lineages.

### 2.2. Reduced Production of IFN-γ by Th1 and Th17 Cells Is Not a Consequence of TNF-α-Mediated Apoptosis

**Figure 4:**
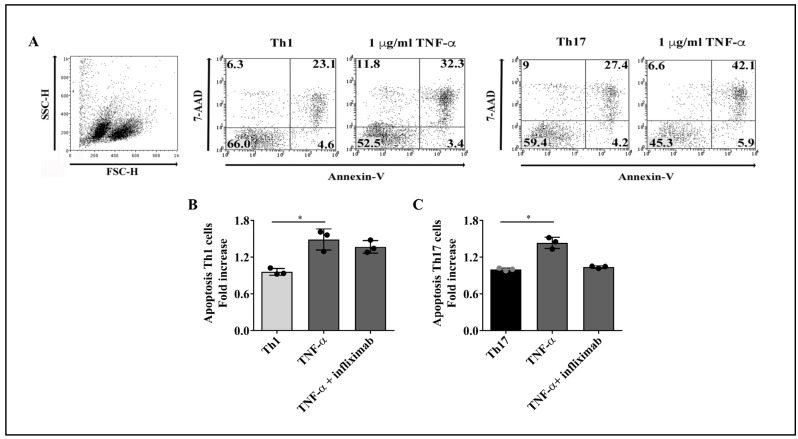
Effect of TNF-α on Th1 and Th17 cells apoptosis. Purified T cell subpopulations were cultured for 4 days with 1 µg/mL of TNF-α alone or in the presence of a TNF-α neutralizing mAb (infliximab), stimulated with PMA and ionomycin plus brefeldin A for 5 h, stained with annexin-V and 7-AAD, and analyzed by flow cytometry. (**A**) Representative dot plots of the gating strategy to assess cell apoptosis. Total purified cells were analyzed. The numbers in each quadrant represent the percentage of cells. The effect of TNF-α on cell apoptosis was detected on Th1 (**B**) and Th17 (**C**) cells obtained from three healthy donors. Bars represent the mean values ± SD. Data were normalized against cells that did not receive TNF-α or TNF-α plus anti-TNF-α treatment. Statistical analyses were performed with Kruskal–Wallis followed by Dunn’s multiple comparison tests. * *p* < 0.05.

Since TNF-α is a pro-apoptotic cytokine [25], we asked whether the TNF-α-induced reduction in the percentages of IFN-γ-producing Th1 and Th17 lineages (Figure 2B and Figure 3B, respectively) was attributable to increased apoptosis. For this purpose, purified T cell subsets were cultured with TNF-α for 4 days, with or without infliximab, and apoptosis was evaluated on total cells, according to cell surface expression of annexin-V and incorporation of 7-AAD (Figure 4A). TNF-α treatment promoted apoptosis at similar levels on Th1 (Figure 4B) and Th17 cells (Figure 4C). Since TNF-α was shown to induce an increment in the proportion of IL-17-producing Th1 and Th17 lymphocytes (Figure 2C and Figure 3C, respectively), the decrease in the frequency of Th1 and Th17 cells producing IFN-γ following TNF-α stimulus may not be a pure consequence of TNF-α-mediated apoptosis of these T cell subsets.

### 2.3. TNF-α Does Not Impact Membrane TNF-α Receptors Levels on Th1 and Th17 Cells

It has been previously demonstrated that CD4^+^ T lymphocytes express both TNF-α receptors, TNFR1 and TNFR2 [24]. Since TNF-α affected IFN-γ and IL-17 production by Th1 and Th17 cells (Figure 2 and Figure 3, respectively), we asked whether the levels of TNF-α receptors vary on the surface of these T cell subsets upon TNF-α stimulus.

**Figure 5:**
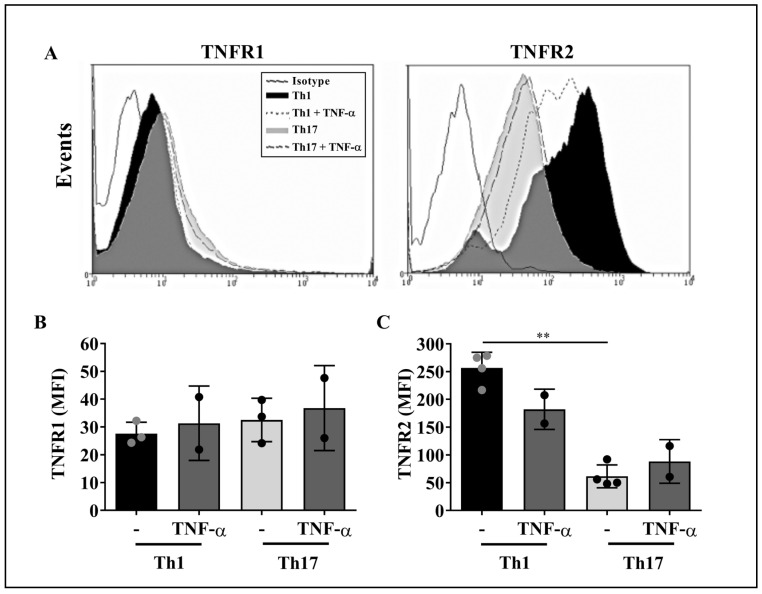
TNF-α does not impact the levels of TNFR1 and TNFR2 on Th1 and Th17 cells. Purified CD4^+^ T cell subsets were stimulated with 1 µg/mL of TNF-α for 24 h and stained, for flow cytometry, with anti-human TNFR1 or TNFR2 mAbs. (**A**) Representative histograms showing the expression levels of TNFR1 and TNFR2 on Th1 and Th17 cells at baseline conditions and upon TNF-α stimulus. Solid black line histograms, isotype control; black histograms, Th1 cells; grey histograms, Th17 cells; dashed line histograms, TNF-α-treated Th1 cells; long dashed line histograms, TNF-α-treated Th17 cells. The levels of TNFR1 (**B**) and TNFR2 (**C**) were measured on purified CD4^+^ T cell subpopulations obtained from two to four healthy controls. Bars represent the mean values ± SD. Statistical analyses were carried out with Kruskal–Wallis followed by Dunn’s multiple comparison tests. ** *p* < 0.01.

Representative flow cytometry histograms of TNF-α receptors expression on purified CD4^+^ T cell subsets are shown in Figure 5A. The analysis of the MFI values revealed that both, Th1 and Th17 cells expressed similar baseline levels of TNFR1, which were not affected by TNF-α stimulus (Figure 5B). In turn, the levels of TNFR2 on Th1 cells were intrinsically higher compared to Th17 cells; such TNFR2 expression pattern did not change upon TNF-α stimulus (Figure 5C). Therefore, it appears that TNF-α does not alter the expression of its cognate receptors on Th1 and Th17 cells.

### 2.4. TNFR2 Mediates the Decrease in IFN-γ Production by Th1 and Th17 Cells

**Figure 6:**
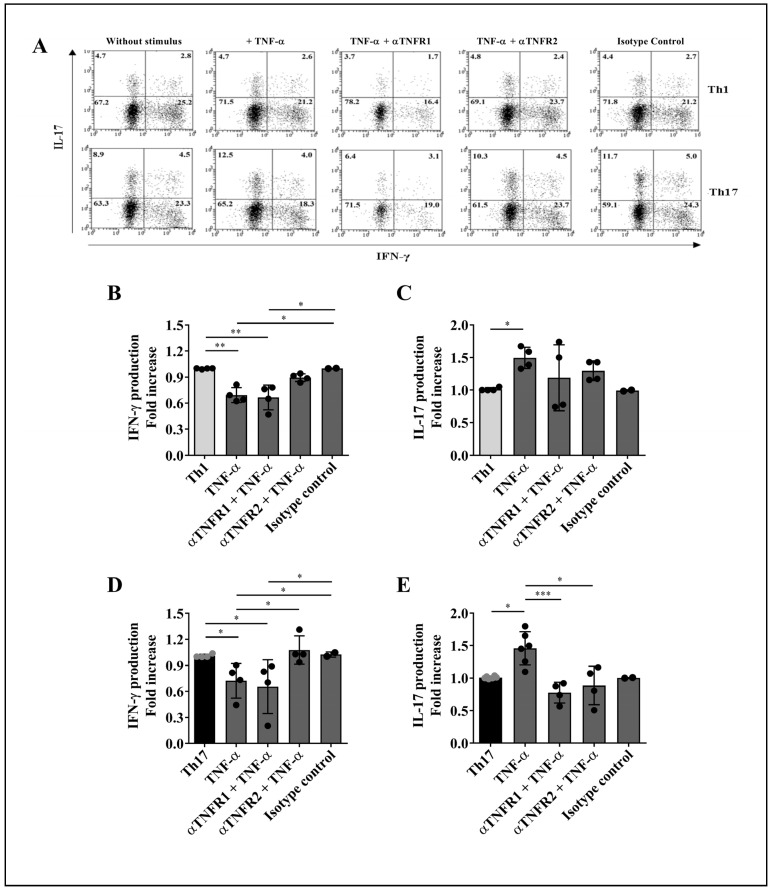
Effect of TNFR1 or TNFR2 blockade on the production of IFN-γ and IL-17 by Th1 and Th17 cells. Purified CD4^+^ T lymphocyte subpopulations were incubated with neutralizing antibodies to TNFR1 or TNFR2 for 1 h prior to a 4-day stimulus with TNF-α (1 µg/mL). Intracellular staining of IFN-γ and IL-17 was assessed by flow cytometry. (**A**) Representative dot plots of Th1 and Th17 cells treated with anti-TNFR1 or anti-TNFR2 in the presence or absence of TNF-α. An isotype control was used to discard non-specific effects of the neutralizing antibodies. The fold increase in the percentages of IFN-γ (**B**,**D**) or IL-17 (**C**,**E**) producers was measured on Th1 and Th17 cells obtained from two to six healthy donors. Bars represent the mean values ± SD. Data were normalized against cells that did not receive treatment with TNF-α or TNF-α plus TNFRs blocking mAbs. For statistical analysis, Kruskal–Wallis and Dunn’s multiple comparison tests were performed. * *p* < 0.05, ** *p* < 0.01, *** *p* < 0.001.

To investigate whether the engagement of distinct TNF-α receptors accounts for the effects of TNF-α on cytokine production by Th1 and Th17 cells, TNFR1 or TNFR2 activation was selectively inhibited on purified lymphocytes using specific neutralizing antibodies prior to stimulation with TNF-α. Cytokine production by both T lymphocyte subsets was evaluated by flow cytometry, as illustrated by the representative dot plots shown in Figure 6A.

TNFR1 blockade did not interfere with TNF-α-mediated reduction in the frequency of IFN-γ^+^ Th1 cells (Figure 6B) or IFN-γ^+^ Th17 cells (Figure 6D). In turn, TNFR2 neutralization prevented the decrease in the percentage of IFN-γ^+^ Th1 cells under TNF-α stimulus (Figure 6B); similarly, TNFR2 inhibition reverted the effect of TNF-α on IFN-γ production by Th17 cells (Figure 6D). Hence, these data suggest that the decrease in IFN-γ production by Th1 and Th17 cells is mediated by TNFR2 signaling, regardless of TNFR1 activation.

### 2.5. Engagement of TNFR1 and TNFR2 Enhances IL-17 Production by Th1 and Th17 Cells

Using the same experimental setting as above, we observed that selective blockade of TNFR1 or TNFR2 restrained the TNF-α-mediated increase in the frequency of Th1 cells producing IL-17 (Figure 6C). Likewise, inhibition of either receptor rescued the basal levels of Th17 cells producing IL-17 (Figure 6E). These results propose that activation of both TNF-α receptors boosts the capacity of Th1 and Th17 cells to produce IL-17.

### 2.6. Altered Expression of TNF-α Receptors on Th1 or Th17 Cells from RA Patients Is Not Modified by Adalimumab Treatment

The levels of TNF-α receptors had not been previously assessed on circulating Th1 and Th17 cells in patients with RA. Thus, we decided to evaluate the expression of TNFR1 and TNFR2 on these T cell subpopulations in RA patients before and after anti-TNF-α therapy with adalimumab and compare it with that of healthy controls. Limitations concerning the amount of blood obtained from patients prevented us to isolate Th1 and Th17 cells based on their cytokine production. Therefore, peripheral Th1 cells were defined by surface expression of CXCR3 on CD4^+^CCR6^−^CD161^−^ T cells, whereas Th17 lymphocytes were identified by the concomitant expression of CCR6 and CD161 on CD4^+^CXCR3^−^ T lymphocytes, as previously reported [34] and indicated in the flow cytometry staining strategy depicted in Supplementary Figure S3.

**Figure 7:**
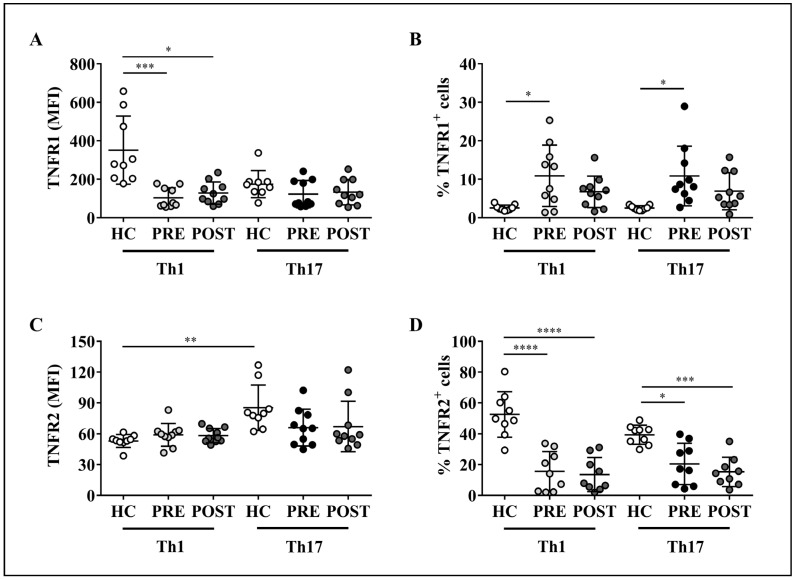
Expression of TNFR1 and TNFR2 on Th1 and Th17 cells present in the peripheral blood of rheumatoid arthritis (RA) patients treated with adalimumab. Cell staining for flow cytometry analysis was performed on PBMC samples from healthy controls (*n* = 9) and RA patients (*n* = 10) before (PRE) and after (POST) treatment with adalimumab. The levels of TNFR1 (**A**) and TNFR2 (**C**) are expressed in MFI values. The frequency of TNFR1 (**B**) and TNFR2 (**D**)-expressing lymphocytes are also shown. Each symbol represents data for one individual. Mean values ± SD are indicated. Significance was assessed with non-parametric Kruskal–Wallis test followed by Dunn’s multiple comparison test (for MFI data) or parametric one-way ANOVA plus Tukey’s post-test (for lymphocyte frequencies data). * *p* < 0.05, ** *p* < 0.01, *** *p* < 0.001, **** *p* < 0.0001.

Our results demonstrate that, when compared to healthy controls, Th1 cells from RA patients displayed significantly lower levels of TNFR1, either before or after treatment with adalimumab, while the levels of this receptor on Th17 cells were similar between controls and patients with or without the biologic therapy (Figure 7A). Interestingly, in healthy controls, the levels of TNFR2 on Th1 cells were lower than on Th17 cells. Such a differential expression pattern was not detected between Th1 and Th17 lymphocytes from RA patients, either before or after adalimumab therapy; in addition, the levels of TNFR2 on both T cell subsets were equivalent between controls and patients before or after therapy (Figure 7C). Of note, the baseline levels of TNFR1 and TNFR2 on both T cell subsets from RA patients were not affected by adalimumab administration (Figure 7A,C, respectively).

On the other hand, RA patients presented higher percentages of TNFR1^+^ Th1 and TNFR1^+^ Th17 cells before, but not after adalimumab treatment, relative to controls (Figure 7B). In contrast, the frequency of TNFR2^+^ Th1 and TNFR2^+^ Th17 cells was significantly lower in patients either before or after anti-TNF-α therapy, as compared to healthy controls (Figure 7D). Importantly, as in the case of TNF-α receptors levels in RA patients, the baseline percentages of Th1 and Th17 lymphocytes expressing TNFR1 and TNFR2 did not change upon therapeutic neutralization of TNF-α (Figure 7B,D, respectively).

Taken together, these results indicate that Th1 and Th17 cells from RA patients exhibit an altered expression pattern of TNFR1 and TNFR2 compared to healthy subjects, and that therapeutic administration of adalimumab to these patients does not restore the levels of TNF-α receptors on the aforementioned cell populations.

## 3. Discussion

Increased levels of TNF-α in the blood and joints of RA patients have been associated with synovial inflammation and bone and cartilage destruction, thus linking this cytokine with the pathogenesis of the disease [35,36]. Although Th1 and Th17 cells have also been shown to act as key regulators in RA, the activity of TNF-α on the phenotype and functions of these effector T lymphocytes still remains to be completely understood.

Here, we studied the response of committed Th1 and Th17 lymphocytes to TNF-α stimulus. Our results showing that TNF-α induces a decrease in the percentage of IFN-γ-producing T cells are in accordance with those obtained by Aspalter et al. [37], who found diminished IFN-γ production by total CD3/CD28-activated T lymphocytes upon TNF-α stimulus. In our experimental settings, enriched CD4^+^ T cells received co-stimulatory stimulus with anti-CD28 and anti-CD49d prior to cell sorting and TNF-α treatment. However, the fact that IFN-γ production by purified Th1 and Th17 cells treated with TNF-α was not affected by other T cell populations may be relevant for a more selective analysis of the effect of TNF-α on individual T lymphocyte subsets.

Not only did TNF-α reduce IFN-γ production by Th1 and Th17 cells; the cytokine was also able to stimulate both T cell subpopulations to produce IL-17. Although several reports indicate that TNF-α does not participate directly in the differentiation of the Th17 cell profile [38,39,40], our results suggest that TNF-α favors peripheral Th17 lineage stability and/or maintenance, possibly by attenuating, on Th1 and Th17 cells, the production of IFN-γ, a potent inhibitor of Th17 cells [17]. Therefore, we hypothesize that the TNF-α-induced decrease in the frequencies of IFN-γ^+^Th1 and IFN-γ^+^Th17 cells participates in the stabilization of a Th17 phenotype, while preventing the development of Th1-like Th17 cells (or IFN-γ-producing Th17 cells) [41], suggesting that TNF-α presents an indirect effect on the persistence of Th17 lymphocytes. In this sense, we have previously demonstrated that treatment of RA patients with adalimumab promoted a significant reduction in the frequency of peripheral Th17 cells [42], thus emphasizing the modulatory role of this cytokine on Th17 cell fitness. Future investigation including IFN-γ blockage in the setting used herein may help test our hypothesis.

TNF-α-mediated induction of IL-17 production by Th17 cells may play a role in the pathogenesis of RA, given that patients with active disease and high levels of TNF-α in the blood present increased levels of IL-17A in the joints and augmented frequency of peripheral and tissue-infiltrating pathogenic Th17 lymphocytes [43,44]. It is important to consider that the purified Th17 cells used in our present study were obtained from healthy donors; thus, these cells may belong to a “conventional”, non-pathogenic Th17 lymphocyte subpopulation. Therefore, it will be interesting to further analyze whether TNF-α also regulates IL-17 production by pathogenic Th17 cells from RA patients in vitro.

The biological effects of the TNF-α/TNF-α receptors axis on autoimmune diseases, including RA, have been intensively explored. Of importance to our current study is the work of Notley et al. [31], who have observed that the proportion of CD4^+^ T cells producing IFN-γ and IL-17 in the lymph nodes of CIA-stimulated mice lacking TNFR1 was significantly higher than in TNFR2-knockout or wild-type mice, indicating that inhibition of Th1 and Th17 responses is mediated by TNFR1. Conversely, in a mouse model that overexpresses TNF-α and develops spontaneous RA-like lesions in the joints, deficiency of TNFR2, but not TNFR1, led to a more aggressive disease, with exacerbated synovial hyperplasia and enhanced destruction of bone and cartilage [32]. Moreover, in a study using an ex vivo-cultured human RA synovial membrane mononuclear cells model, the authors observed that specific blockade of TNFR1 resulted in significant inhibition of pro-inflammatory cytokine and chemokine production [45]. Accordingly, in a murine model of erosive arthritis, the selective absence of TNFR1 on hematopoietic cells attenuated bone destruction, while the absence of TNFR2 on these cells increased joint inflammation and erosive bone destruction [33]. These and other evidence [46] thus propose that the two TNF-α receptors contribute to the pleiotropic nature of this cytokine, as TNF-α-induced pro-inflammatory functions are predominantly mediated by TNFR1, while TNFR2 provides a more immunoregulatory landscape in some diseases. Therefore, experimental settings targeting either TNFR1 or TNFR2 may reveal distinctive effects of TNF-α on particular cells which, otherwise, would be overlooked under overall TNF-α blockade.

In this sense, here we observed that, constitutively, Th1 and Th17 cells presented similar levels of TNFR1, whereas TNFR2 expression was higher on Th1 cells compared to Th17 lymphocytes. Interestingly, although TNF-α did not exert a significant effect on the levels of both receptors on the membrane of T cell subsets compared to their baseline expression, this apparent lack of effect does not exclude the possible impact of this cytokine on its cognate receptors’ activation thresholds and/or the triggering of specific intracellular signaling pathways on distinct T cell subpopulations.

Indeed, our results demonstrate that, under TNF-α stimulation, selective blockade of TNFR2, but not TNFR1, reestablished the baseline proportions of IFN-γ^+^ Th1 and IFN-γ^+^ Th17 cells observed in the absence of TNF-α treatment, suggesting that TNFR2 signaling regulates the TNF-α-induced decrease in the frequency of Th1 and Th17 cells producing IFN-γ. It is important to mention that, even though TNFR2 full activation is mediated by membrane-bound TNF-α [47], we cannot rule out the participation, in our assays, of other soluble molecules that can also trigger the activation of this receptor. Such is the case for lymphotoxin (LT)-α (formerly known as TNF-β), the closest homolog of TNF-α that also acts as an agonist to TNFR1 and TNFR2 [48], and which is produced by Th1 cells [49]. As LT-α has also been shown to contribute to the pathogenesis of RA [50], it would be valuable to further investigate the effect of TNF-α on the functions and production of LT-α by T cell subsets in RA patients.

As for IL-17, inhibition of either TNF-α receptor individually, in the presence of exogenous TNF-α, restored the baseline proportions of Th1 and Th17 cells producing IL-17, indicating that engagement of TNFR1 and TNFR2 is necessary to achieve the TNF-α-mediated increase in IL-17 production by these T cell subsets. Since neutralization of TNF-α was effective at re-establishing the baseline frequencies of Th1 and Th17 cells that produce IFN-γ and IL-17, blockade of either TNF-α or TNFR2 may be sufficient to revert the TNF-α-induced alterations in cytokine production by these T cell lineages.

Previous reports have shown that total PBMCs from patients with RA present higher levels of TNFR1 mRNA [27], as well as an increased percentage of peripheral CD4^+^ T cells expressing TNFR1 [51]. In turn, the levels of TNFR2 mRNA were found to be equivalent to those of healthy controls [27]; accordingly, TNFR2 expression on peripheral CD4^+^ T cells did not differ between RA patients and healthy donors [51]. Partially in line with these studies, our results showed that, even though Th1, but not Th17 cells from RA patients presented lower levels of TNFR1, the frequencies of TNFR1^+^ Th1 and TNFR1^+^ Th17 cells were found to be significantly increased in RA patients compared to healthy controls. In contrast, the percentages of Th1 and Th17 cells expressing TNFR2 were decreased in RA patients compared to controls, although the levels of TNFR2 on these T cell subsets were similar between both groups of individuals.

The decreased expression of TNFR1 on Th1 cells and the lower frequency of TNFR2^+^ Th1 and TNFR2^+^ Th17 cells may be explained by the well-known mechanism of receptor internalization, since stimulation with TNF-α can lead to TNFR1 and TNFR2 endocytosis and further inhibition of their long-term functions [52,53]. However, the expression profile of TNF-α receptors on the analyzed T cell subsets was sustained in RA patients receiving adalimumab, as patients’ basal levels and percentages of Th1 and Th17 cells expressing either TNFR1 or TNFR2 were not altered by therapeutic neutralization of TNF-α. Therefore, the possibility that LT-α is promoting TNF-α receptors downregulation in these patients may help interpret these results, as adalimumab does not bind to LT-α [54].

Alternatively, the proteolytic shedding of membrane-bound TNF-α receptors ectodomains by the metalloprotease TNF-α converting enzyme (TACE, also known as ADAM17) [24,55], resulting in the release of soluble TNF-α binding proteins and reduction in cellular responses to TNF-α [56], may also play a part on the decreased levels of TNF-α receptors on T lymphocyte subpopulations in RA patients. Indeed, patients with acute RA have shown increased serum levels of soluble TNFR1 and TNFR2 compared to healthy controls [57,58], which may correlate inversely with the levels of TNF-α receptors on the membrane of diverse circulating immune cells. Moreover, based on reports demonstrating that high levels of TNFR1, but not TNFR2 are detected on CD4^+^ T lymphocytes that migrate to rheumatoid synovium [51], it would be interesting to analyze the expression of TNF-α receptors on Th1 and Th17 cells infiltrating the synovia of RA patients before and after therapeutic intervention with TNF-α inhibitors. Nevertheless, we cannot rule out the fact that the reduction in the levels of TNF-α receptors on peripheral T helper subsets may or may not compromise their functionality, which poses new challenges to uncover the biological significance of our observations.

In this sense, our study is subject to some limitations. First, we only evaluated the frequency of Th1 and Th17 cells producing IFN-γ and IL-17 by flow cytometry. Although such an approach does not reveal the capacity of these cells to secrete the cytokines analyzed, which would adequately reflect their ability to affect other cells, intracellular cytokine staining provides the possibility to identify which specific cells are producing the cytokine of interest. Here, we aimed at assessing IFN-γ and IL-17-only producers within each isolated T cell subtype, and this can only be achieved on a single-cell basis (i.e., by flow cytometry). Second, although we found no change in the expression of TNF-α receptors on Th1 and Th17 cells in RA patients before and after adalimumab treatment, we did not evaluate whether this conserved phenotype relates to T cell effector functions in these patients. Moreover, our study only focused on the effects of TNF-α treatment and TNF-α receptors blockade on cytokine production by peripheral T lymphocytes derived from healthy donors. Since RA patients present T cells with altered functionality, which contributes to the pathogenesis of RA [59], we believe that the same evaluation performed herein, this time on RA patients’ samples, would greatly enhance the understanding of the role of TNFR1 and TNFR2 in this disease.

Although the intervention of RA patients with adalimumab induces rapid and sustained improvements in disease activity and ameliorates physical function [60], a significant percentage of patients under adalimumab treatment either do not respond adequately or relapse within 12 months of treatment [61]. Moreover, an association between the lack of response to TNFα neutralization and high levels of IL-17-producing T cells before treatment, along with an increased frequency of Th17 cells after adalimumab treatment, has been demonstrated [62]. Since blockade of TNFR2 in the presence of exogenous TNF-α restores the basal levels of IFN-γ and IL-17-producing Th1 and Th17 cells, as observed herein, we believe that targeting TNFR2 with a blocking antibody may represent an alternative strategy to normalize the levels of RA-associated cytokines, thus preventing the harmful effects of TNF-α in this disease, especially in patients that do not respond adequately to TNF-α antagonists.

Nevertheless, it is important to bear in mind that stimulation of TNFR2 with agonist antibodies has been shown to promote proliferation and activation of regulatory T cells (Tregs) with immunosuppressive function [23]. In addition, anti-TNFR2 agonist antibodies tested in clinical trials have shown promising therapeutic potential for autoimmune diseases, such as type 1 diabetes [63]. Thus, it would be valuable to test the effect of TNFR2 blockade on Tregs and other CD4^+^ T cell subpopulations and on the levels of cytokines they produce to potentially uncover biological mechanisms that may help develop novel therapeutic strategies to treat RA or other autoimmune diseases.

In conclusion, the data presented herein suggest that, on one hand, TNF-α affects the phenotype of Th1 and Th17 lymphocytes by decreasing their production of IFN-γ through TNFR2 activation and by stimulating IL-17 synthesis through TNFR1 and TNFR2 signaling, thus proposing that TNFR2 could represent an interesting target to normalize the levels of IFN-γ and IL-17 produced by Th1 and Th17 cells. Hence, TNFR2 neutralization may be taken into consideration when designing TNFR-based biologicals to antagonize the harmful effects of TNF-α in RA. Not only can TNF-α participate in the processes of perpetuation and chronicity of this disease, but also in its upstream pathogenic mechanisms, actively modulating the effector functions of key subsets of T cells through differential actions on its receptors.

## 4. Materials and Methods

### 4.1. Healthy Donors and RA Patients

Nine healthy donors (8 female and 1 male) aged 48 ± 7 years (range, 41–59 years) and ten female RA patients aged 52 ± 11 years (range, 24–65 years) were included in this study. Patients were recruited at the Hospital Clínico, Universidad de Chile. At the time of study entry, patients presented with RA duration of 15 ± 7 years (range, 1.5–26 years), and none of them had received biologic disease-modifying anti-rheumatic drugs. RA characterization was defined according to the American College of Rheumatology (ACR) criteria [64]. All patients exhibited an active disease as defined by a disease activity score for 28 commonly involved joints in RA (DAS28) > 5.1 (range, 5.1–7.8). Patients received a total of 4 subcutaneous doses of 40 mg/mL of adalimumab (kindly provided by Abbott Laboratories, USA) every two months for 8 months. Peripheral blood samples were collected from patients prior to drug administration and 6 months after beginning treatment.

### 4.2. Buffy Coats, Blood Samples and PBMC

Buffy coats from healthy volunteers were provided by the Hospital Clínico, Universidad de Chile. Peripheral blood samples from RA patients and controls were used for PBMCs isolation by density gradient centrifugation using Ficoll-Hypaque (GE Health Care, Chicago, IL, USA). PBMCs were cryopreserved in liquid nitrogen in fetal bovine serum (FBS) (HyClone, Logan, UT, USA) and 10% dimethyl sulphoxide, until use.

### 4.3. Cell Sorting

Buffy coats from healthy donors were used to enrich CD4^+^ T cells by negative selection using the RosetteSep™ Human CD4^+^ T cell enrichment Kit (Stem Cell Technologies, Vancouver, BC, Canada), according to the manufacturer’s instructions. Enrichment of CD4^+^ cells was assessed by flow cytometry using a PECy7-conjugated anti-CD4 mAb (clone OKT4), and antibody specificity was validated with a PECy7-conjugated mouse IgG2b, κ isotype control (both from BioLegend, San Diego, CA, USA). For the isolation of specific cytokine-producing T cells, cytokine capture assays were performed [65]. For this purpose, enriched CD4^+^ T cells were stimulated with soluble anti-CD28 (0.5 mg/mL) and anti-CD49d (1 mg/mL) mAbs (both from Thermo Fisher Scientific, Waltham, MA, USA) plus 15 ng/mL of PMA and 1 µg/mL of ionomycin (both from Sigma-Aldrich, St. Louis, MO, USA) for 3 h and 30 min at 37 °C and 5% CO_2_ to induce cytokine production. Cells were washed with 1% FBS in phosphate-buffered saline (PBS) and incubated in the dark, for 12 h at 4 °C, with anti-CD45-IFN-γ-biotin-avidin or anti-CD45-IL-17-biotin-avidin antibody conjugates (both from Thermo Fisher Scientific) to obtain IFN-γ and IL-17-secreting CD4^+^CD45^+^ T cells, respectively. Cells were then stained with anti-IFN-γ-FITC and anti-IL-17-PE mAbs (both from Thermo Fisher Scientific) for 30 min at 4 °C in the dark for further Th1 and Th17 cell sorting, respectively, using a BD FACSAria III cell sorter (BD Biosciences, San Jose, CA, USA).

### 4.4. Cell Staining and Flow Cytometry for Transcription Factors and Intracellular Cytokines

The purity of sorted Th1 and Th17 lymphocytes was evaluated by flow cytometry following cell permeabilization with the BD Cytofix/Cytoperm™ kit (BD Biosciences) for 20 min plus intracellular staining with anti-T-bet-PerCP/Cy5.5 and anti-RORγt-APC mAbs (both from Thermo Fisher Scientific) for 30 min at 4 °C in the dark. Cells were then analyzed by flow cytometry with a FACScalibur flow cytometer (BD Biosciences).

To evaluate the effect of TNF-α on intracellular cytokine production, Th1 and Th17-sorted cells were resuspended and distributed, at a density of 7.5 × 10^4^ cells/well in 200 µL of X-Vivo 20 medium (Lonza, Basel, Switzerland), in flat-bottomed 96-well plates and cultured for 4 days, at 37 °C and 5% CO_2_, with recombinant human TNF-α (1 µg/mL) (Thermo Fisher Scientific) in the presence or absence of an anti-TNF-α blocking mAb (infliximab) (10 µg/mL) (Centocor, Inc. (Horsham, PA, USA), Schering-Plough, Kenilworth, NJ, USA). Next, cells were stimulated with 15 ng/mL of PMA and 1 µg/mL of ionomycin plus 10 µg/mL of brefeldin A (Thermo Fisher Scientific) for 5 h, after which they were harvested, washed, permeabilized with a fixation and permeabilization kit (Thermo Fisher Scientific), and incubated with anti-IFN-γ-FITC and anti-IL-17-PE mAbs for 30 min at 4 °C in the dark. Cells were further analyzed by flow cytometry using a FACScalibur flow cytometer. Unstained cells were acquired to control for the absence of carryover fluorescence from the cell sorting procedure.

All data from cell sorting and flow cytometry were acquired with the CellQuest program (BD Biosciences) and evaluated with the Weasel Software (Walter and Eliza Hall Institute of Medical Research, WEHI, Parkville, VIC, Australia). The lymphoid population was defined according to forward and side scatter patterns. In all experimental settings, dead cells were excluded from analysis using the LIVE/DEAD^®^ staining kit (ThermoFisher Scientific).

### 4.5. Apoptosis Assay

To determine TNF-α-mediated apoptosis in cell cultures, Th1 and Th17-sorted lymphocytes were resuspended and cultured, for 4 days, with TNF-α (1 µg/mL) in the presence or absence of infliximab (10 µg/mL). Cells were then labeled with PE-conjugated annexin-V and 7-AAD (both from Thermo Fisher Scientific), and cell viability was immediately determined by flow cytometry.

### 4.6. Flow Cytometry for TNF-α Receptors

To evaluate the levels of TNFR1 and TNFR2 on the cell membrane, in the presence of TNF-α, purified Th1 and Th17 lymphocytes were maintained for 24 h in culture medium prior to a 24 h stimulus with 1 µg/mL of TNF-α. Cells were then stained with a mouse anti-human TNFR1-APC mAb (clone 16803) (R&D Systems, Minneapolis, MN, USA) and a rat anti-human TNFR2-PE mAb (clone hTNFR-M1) (BD Biosciences) for 30 min at 4 °C in the dark. A mouse IgG1κ mAb (clone P3.6.2.8.1) and a rat IgG2bκ mAb (clone eB149/10H5) (both from ThermoFisher Scientific) were used to test anti-TNFR1 and anti-TNFR2 mAbs specificity, respectively. Cells were further analyzed by flow cytometry, as previously described.

### 4.7. Blockade of TNF-α Receptors

For TNF-α receptors blockade assays, purified Th1 and Th17 cells were incubated with 5 ng/mL of either an unconjugated mouse anti-human TNFR1 mAb (clone 16803) or an unconjugated mouse anti-human-TNFR2 (clone 22210) (both from R&D Systems) for 1 h. An IgG1κ isotype control (clone 11711) (R&D Systems) was used to rule out non-specific effects of the neutralizing antibodies. Cells were then stimulated with 1 µg/mL of TNF-α for 4 days, after which intracellular staining of IFN-γ and IL-17, followed by analysis by flow cytometry, were performed.

### 4.8. Ex Vivo Staining of PBMCs from RA Patients

To analyze the levels of TNFR1 and TNFR2 on the membrane of Th1 and Th17 cells present in the blood of RA patients treated with adalimumab, PBMCs from RA patients and healthy donors were incubated with a mouse anti-human TNFR1-APC mAb (clone 16803) (R&D Systems), a rat anti-human TNFR2-PE mAb (clone hTNFR-M1) (BD Biosciences), a mouse anti-human CD161-FITC mAb (clone HP-3G10), a mouse anti-human CCR6-PerCP/Cy5.5 mAb (clone G034E3), and a mouse anti-human CXCR3-APC/Cy7 mAb (clone G025H7) (all from BioLegend) for 30 min at 4 °C in the dark. TNF-α receptors levels were evaluated, by flow cytometry, on CD4^+^CCR6^-^CXCR3^+^CD161^-^ Th1 cells and CD4^+^CCR6^+^CXCR3^-^CD161^+^ Th17 lymphocytes.

### 4.9. Statistical Analysis

Normality was evaluated by the Kolmogorov–Smirnov test. Significance was determined using either the Mann–Whitney test, Kruskal–Wallis test with Dunn’s multiple comparison post hoc test, or one-way ANOVA with Tukey’s multiple comparison test, all with a confidence interval of 95%. *P*-values lower than 0.05 were considered statistically significant. For all analysis, GraphPad Prism v7.04 (GraphPad Software, San Diego, CA, USA) was employed.

## Data Availability

Not applicable.

## Supplementary Materials

The following supporting information can be downloaded at: https://www.mdpi.com/article/10.3390/ijms23169306/s1.
