# Supplementary material for: TNF-α Affects Signature Cytokines of Th1 and Th17 T Cell Subsets through Differential Actions on TNFR1 and TNFR2
Source: Int J Mol Sci. 2022 Aug 18;23(16):9306. doi: 10.3390/ijms23169306 (PMC9408897; doi:10.3390/ijms23169306)
Supplement: Supplementary file 1 [file ijms-23-09306-s001.zip › ijms-1791498-supplementary.pdf]

Supplementary Material

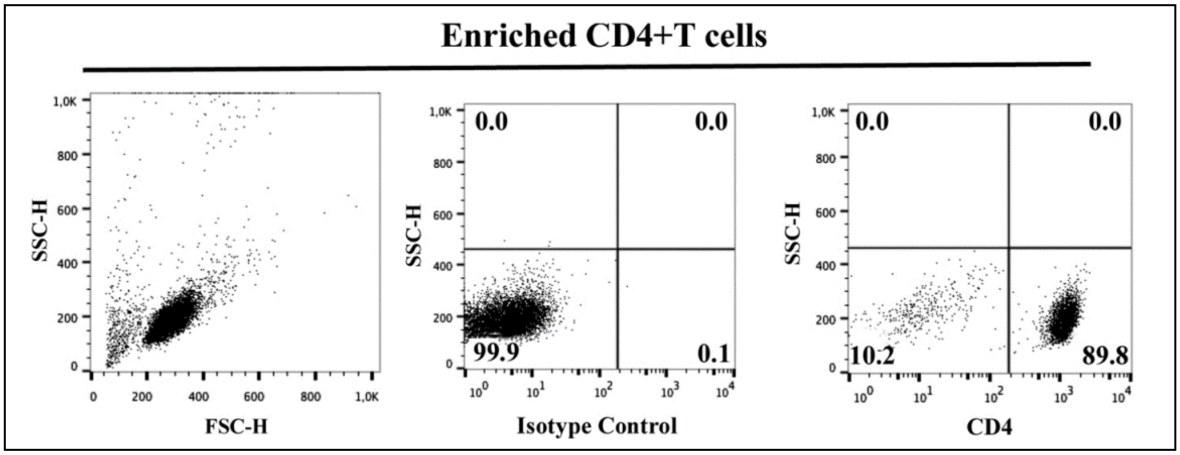

Figure S1. Gating strategy to assess the isolation of peripheral blood CD4$^+$ T helper lymphocytes by flow cytometry. CD4$^+$ T cells were enriched from PBMCs by negative selection using a RosetteSep™ kit (Stem Cell Technologies). The representative dot plots of the forward (FSC-H) and side (SSC-H)-scatter properties of enriched CD4$^+$ T cells are from a healthy donor. Cell purity was detected with an anti-CD4 mAb. Antibody specificity was confirmed with an isotype control.

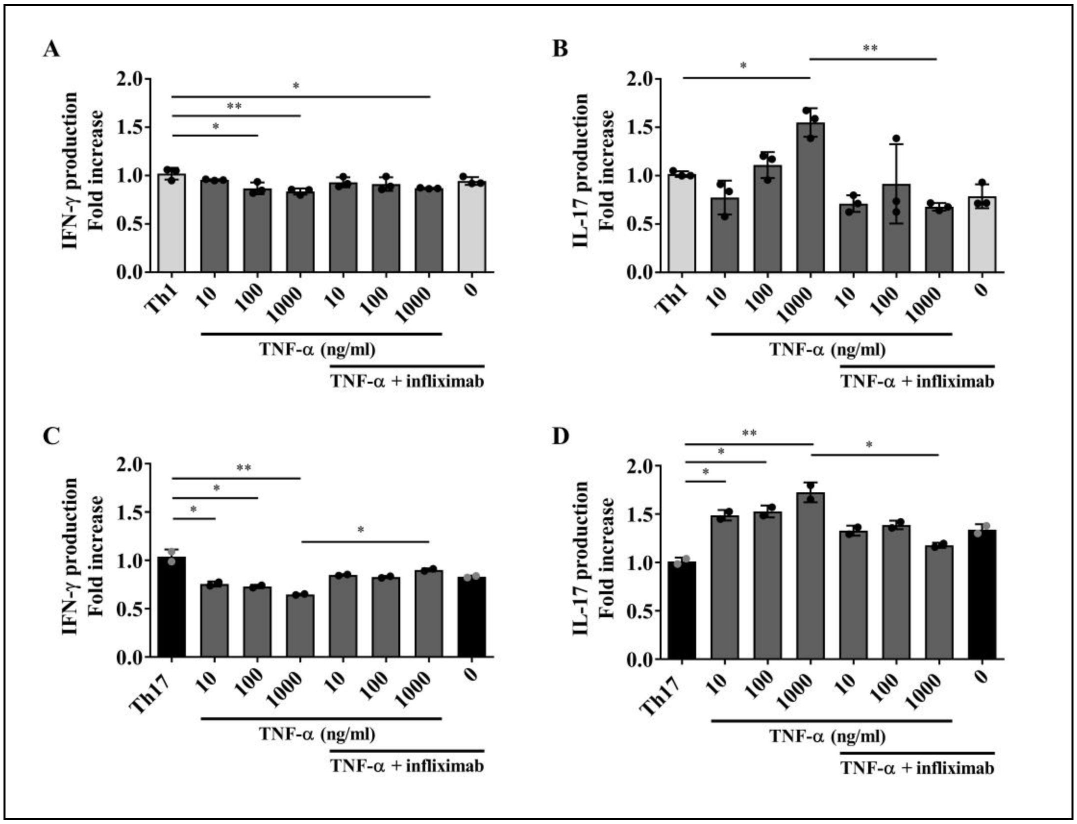

Figure S2. Effect of TNF-α on IFN-γ and IL-17 production by Th1 and Th17 lymphocytes. Sorted T lymphocyte subsets were cultured for 4 days with increasing concentrations of recombinant human TNF-α (0, 10, 100, or 1000 ng/ml) alone or in the presence of an anti-TNF-α blocking mAb (infliximab). Cells were stimulated with PMA and ionomycin plus brefeldin A for 5 h and analyzed for intracellular cytokine production by flow cytometry. IFN-γ (**A**) and IL-17 (**B**) production was measured on Th1 cells from three healthy donors; IFN-γ (**C**) and IL-17 (**D**) were detected on Th17 cells from two healthy controls. Bars represent the mean values ± SD. Data were normalized against cells that did not receive treatment with TNF-α or TNF-α plus infliximab. Statistical analyses were performed with Kruskal-Wallis followed by Dunn's multiple comparison tests. * $p < 0.05$, ** $p < 0.01$.

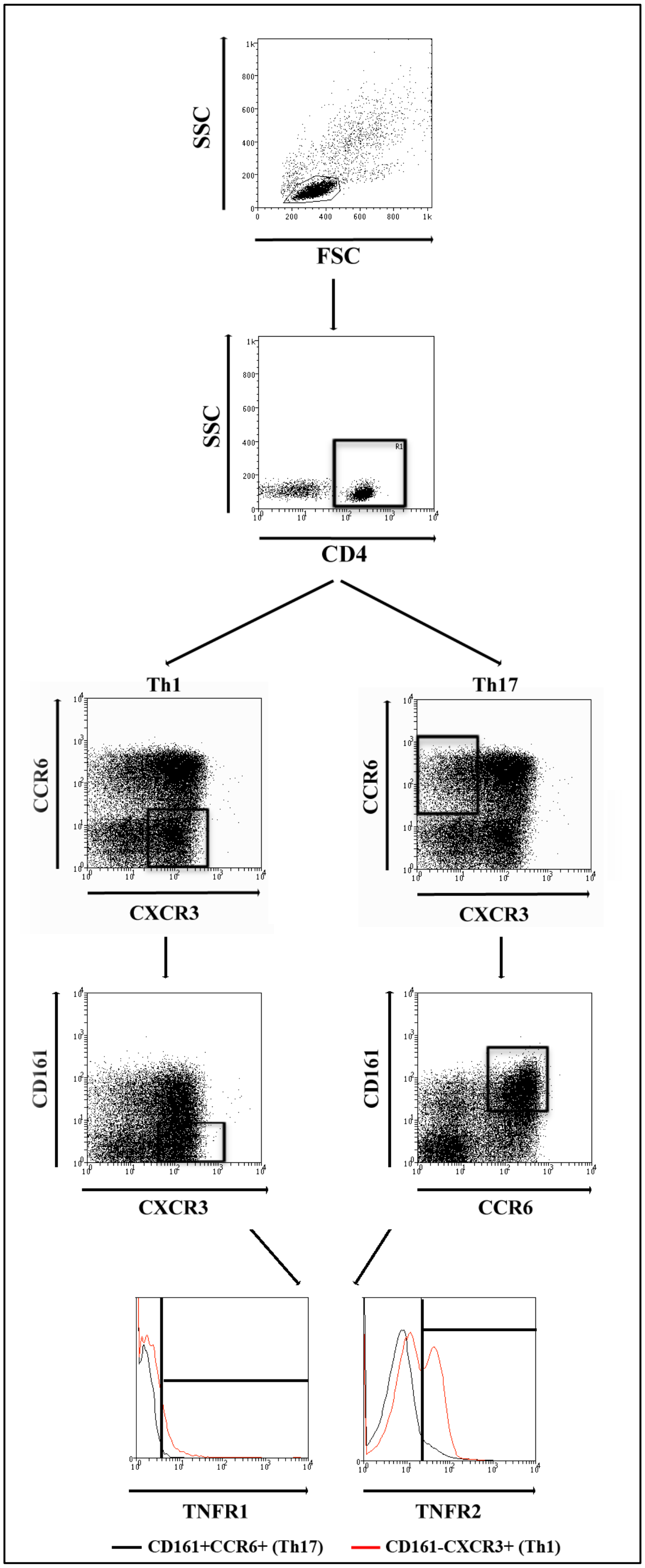

SSC
FSC
SSC
CD4
Th1
Th17
CCR6
CXCR3
CCR6
CXCR3
CD161
CXCR3
CD161
CCR6
TNFR1
TNFR2
CD161+CCR6+ (Th17)
CD161-CXCR3+ (Th1)

Figure S3. Gating strategy to analyze the expression of TNF-α receptors on peripheral blood Th1 and Th17 cells by flow cytometry. The dot plots and histograms represent staining of PBMCs from a healthy control. TNFR1 and TNFR2 expression was evaluated on $CD4^+CCR6^-CXCR3^+CD161^-$ (Th1) cells and $CD4^+CCR6^+CXCR3^-CD161^+$ (Th17) cells.
